# Early-Age Strength of Ultra-High Performance Concrete in Various Curing Conditions

**DOI:** 10.3390/ma8085261

**Published:** 2015-08-24

**Authors:** Jong-Sup Park, Young Jin Kim, Jeong-Rae Cho, Se-Jin Jeon

**Affiliations:** 1 Structural Engineering Research Institute, Korea Institute of Civil Engineering and Building Technology, Goyang-si, Gyeonggi-do 411-712, Korea; E-Mails: jspark1@kict.re.kr (J.-S.P.); yjkim@kict.re.kr (Y.J.K.); chojr@kict.re.kr (J.-R.C.); 2 Department of Civil Systems Engineering, Ajou Univeristy, Suwon-si, Gyeonggi-do 443-749, Korea

**Keywords:** Ultra-High Performance Concrete, curing, strength, cast-in-place

## Abstract

The strength of Ultra-High Performance Concrete (UHPC) can be sensitively affected by the curing method used. However, in contrast to the precast plant production of UHPC where a standard high-temperature steam curing is available, an optimum curing condition is rarely realized with cast-in-place UHPC. Therefore, the trend of the compressive strength development of UHPC was experimentally investigated in this study, with a focus on early-age strength by assuming the various curing conditions anticipated on site. Concrete specimens were cured under different conditions with variables including curing temperature, delay time before the initiation of curing, duration of curing, and moisture condition. Several conditions for curing are proposed that are required when the cast-in-place UHPC should gain a specified strength at an early age. It is expected that the practical use of UHPC on construction sites can be expedited through this study.

## 1. Introduction

Ultra-High Performance Concrete (UHPC) has been one of the most active research fields of concrete recently, because it can contribute to the longer life and economic efficiency of structures [1,2]. Recent extensive studies carried out on UHPC have further improved the quality of high strength or high performance concrete [2,3,4]. While UHPC has superior mechanical properties in terms of compressive and tensile strengths, ductility, and toughness, as well as high flowability and durability, strict quality control is required to ensure these target standards for its production. The term UHPC usually covers various types of cementitious composite materials developed for similar purposes. Similarly to ordinary concrete, UHPC can be either fabricated as precast members at a plant or cast in-place at a construction site. Apart from fully cast-in-place UHPC structures, although a precast-type UHPC structure is planned for stable quality control and acceleration of construction [5], some components, such as the joints of precast UHPC segments, still need to be cast in-place [6]. These cast-in-place components of a UHPC structure can significantly affect the overall quality of the structure and construction speed, since they are cast possibly under limited and unfavorable conditions on the site. Another important application of cast-in-place UHPC is the rehabilitation of existing structures [1,7].

The quality of cast-in-place UHPC can be considerably affected by the mixing, placing, and curing methods used. Some projects have been conducted to extend the use of UHPC to the field, focusing on cast-in-place technologies [1,7,8]. One of the difficulties of casting the UHPC on site is the need for a specially-designed mixer for UHPC that is usually used in a laboratory or a plant. As one of the strategies to cope with this problem, a portable mixer optimized for the UHPC mixture has been developed because the quality of the UHPC using a conventional mixer may be subject to large variation [8]. Another important factor that affects the quality of cast-in-place UHPC is the curing method. Possible curing methods on site may differ from those of precast UHPC segments fabricated in the ideal conditions of a plant. Generally, in precast UHPC, a standard steam curing method is adopted to obtain rapid strength development. However, in cast-in-place UHPC, which is the main focus of this study, curing methods are often limited in terms of curing temperature, curing period, and moisture condition.

Several studies have focused on determining whether a specified compressive strength of UHPC can be attained at 28 days under normal moist curing without heat treatment [1,3,7]. In some cases, however, the specified strength needs to be obtained within an earlier age of UHPC to accelerate construction speed. Therefore, this study presents experimental results on the characteristics of the early-age strength development of UHPC in various curing conditions conceivable at the site. Regarding the terminology for the early age, there is not a clear definition of how short the early age of concrete is. However, 7-day compressive strength, which was determined as the focus of this study by referring to the days required for standard steam curing in plant production, can be regarded as the early-age strength in comparison to 28-day strength that is usually adopted for the design purposes. Factors considered in the experimental program include curing temperature, delay time before the initiation of curing, duration of curing, and moisture condition. The strengths were compared with those of the specimens cured by standard high-temperature steam. Through the analysis of the test results, several requirements for curing are proposed that are required when the specified strength of UHPC should be attained in an early age even though it is cast in-place.

## 2. Curing of UHPC

### 2.1. Development of K-UHPC

**Figure 1:**
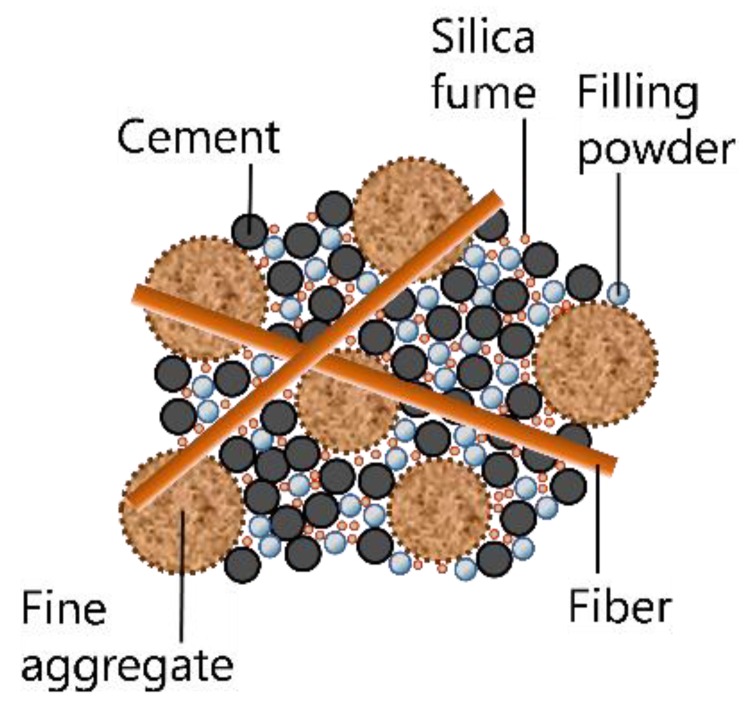
Composition of K-Ultra-High Performance Concrete (K-UHPC) (not to scale).

For the mix design of UHPC, the superior mechanical properties need to be considered, such as strength, ductility, and toughness, in addition to the high flowability of fresh concrete and durability. Therefore, various mix proportions of UHPC have been proposed, so far, depending on the target properties. The K-UHPC developed by the Korea Institute of Civil Engineering and Building Technology is the main focus in this study [4]. Figure 1 shows the schematic composition of K-UHPC and Table 1 presents the specific mix proportion of the K-UHPC which is expressed as a ratio of mass. The mixture consists of cement, silica fume, filling powder, fine aggregate, shrinkage reducing agent, expansive agent, superplasticizer, and steel fibers. Coarse aggregates are not included in the mixture. The cement in Table 1 is Ordinary Portland cement. Silica fume used for K-UHPC requires the specific surface area of more than 150,000 cm^2^/g and SiO_2_ content of more than 96%. The filling powder has the average particle size of 10 μm and SiO_2_ content of more than 96%. Fine aggregate used for K-UHPC is limited to silica sand with the diameter of less than 0.5 mm. The glycol-based shrinkage reducing agent and calcium sulfa aluminate-based expansive agent are added to cope with shrinkage of concrete; in particular, the autogenous shrinkage that is induced by self-desiccation when the water-to-binder ratio is adjusted to be very low to attain high strength. Also, polycarboxylic acid-based superplasticizer is used to ensure high flowability even with a very low water-to-binder ratio. The steel fibers have the diameter of 0.2 mm and the tensile strength of more than 2,000 MPa. The length of the fibers can be chosen among 13, 16 and 20 mm depending on the required tensile characteristics. The binder in the water-to-binder ratio in Table 1 indicates cement plus silica fume. The design recommendations for K-UHPC [4] provide detailed information on each material.

**Table 1:** Mix proportion of K-Ultra-High Performance Concrete (K-UHPC) (ratio of mass).

Item	Value	Item	Value	Item	Value
Water-to-binder ratio	0.2	Filling powder	0.3	Expansive agent	0.075
Cement	1	Fine aggregate	1.1	Superplasticizer	0.018
Silica fume	0.25	Shrinkage reducing agent	0.01	Steel fiber (volume fraction)	1.5%–2%

The specified compressive strength, cracking strength, and tensile strength of K-UHPC are as high as 180, 9.5 and 13 MPa, respectively. In order to ensure these target strengths, initial curing and high-temperature steam curing are recommended in sequence [4]. The initial curing is maintained at 20 °C for 12–48 h, with 24 h recommended, immediately after casting. The following high-temperature steam curing is performed at 90 °C for 24–72 h, typically, with 48 h recommended. It was confirmed that strengths that exceed the specified strengths can be obtained even at an early age immediately after curing if the above curing criteria are fulfilled. These criteria were introduced by taking into consideration the curing of precast members. However, when the K-UHPC is cast in-place, the criteria are barely met in many cases, as a result of the difficulty in controlling the temperature and moisture due to the limited circumstances of the site. This study focuses on the minimum curing conditions of cast-in-place K-UHPC (in circumstances where standard steam curing is not available on site) that are required to ensure a similar target strength to that of precast K-UHPC at an early age. Other properties of K-UHPC, such as shrinkage behavior, including the dominant autogenous shrinkage, were investigated in the previous studies [9].

### 2.2. Previous Studies

Since the concept of concrete maturity was established by Carino *et al.* [10], the importance of curing temperature and age in strength development has been generally accepted. This is the reason why the high-temperature steam curing is preferred in a precast concrete plant. In UHPC, however, an additional closer relationship exists between curing temperature and strength than that in normal concrete, because most UHPC includes a large amount of silica fume due to the various advantageous characteristics [2,11,12], such as a considerable strength increase. Silica fume is transformed to calcium silicate hydrate by reacting with calcium hydroxide through the pozzolanic reaction. This type of reaction tends to be substantially activated under a high temperature [2,11], which is why it is recommended for most UHPC to be cured under a high temperature to ensure rapid strength development. On the other hand, the moisture condition of UHPC containing silica fume should be given special attention in order to cope with the dominant self-desiccation [1,11]. The Silica Fume Association recommends moist curing of the concrete containing the silica fume for at least 7 days [12]. Based on these previous studies, the control of curing temperature and moisture condition would have a crucial effect on the strength development of cast-in-place UHPC.

However, it is usually difficult to apply an ideal curing scheme in terms of temperature and moisture when the UHPC is cast on site, because the construction site has an inferior condition to a laboratory or a precast concrete plant; a realistic curing scheme should, thus, be devised on site. Some researchers have focused on determining whether the UHPC can attain the specified compressive strength at 28 days when subjected to ambient or room temperature and sufficient moisture for a certain period [1,3]. However, sometimes the specified strength needs to be ensured within a shorter period in order to advance the completion date of the structure, even under inferior site conditions, as investigated in this study.

**Figure 2:**
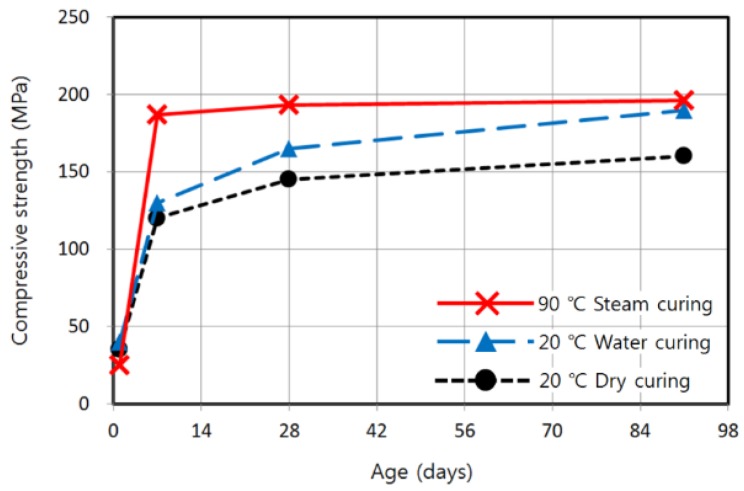
Compressive strength development of K-UHPC with different curing conditions [13].

Ishii *et al.* [5] demonstrated that the early-age strength of the UHPC that they applied to a pedestrian bridge was reduced from 215 to 147 MPa when the steam curing temperature was lowered from 90 to 70 °C. Koh *et al.* [13] reported the results of 20 °C curing of K-UHPC as shown in Figure 2. It was observed that 190 MPa was attained at 91 days under moist curing, which is similar to the strength expected immediately after 90 °C steam curing, while only 60% of 190 MPa was attained at 7 days. In comparison, only 80% of 190 MPa was achieved even at 91 days under a dry condition, demonstrating the importance of moisture in curing. Ahlborn *et al.* [14] reported that the strength of UHPC without the 90 °C steam curing was reduced to 69% and 83% of the specified strength at 7 and 28 days, respectively. Additionally, they studied the effect of delay time before the steam curing and concluded that a delay time of even as long as 10 or 24 days did not significantly affect the strength after the steam curing. Schachinger *et al.* [15] analyzed the degree of hydration using nuclear magnetic resonance and strength of UHPC in terms of curing temperatures ranging from 20 to 90 °C. They showed that the gradual increase of the degree of hydration of silica fume in the specimen cured at a relatively low temperature delayed the specified strength development by as much as several years. Nakayama *et al.* [16] also showed that the early-age strength of UHPC decreased as the curing temperature lowered. Matsubara *et al.* [6] applied a heating system that can realize 40 or 60 °C during curing of the cast-in-place joint components of a precast UHPC. The precast UHPC used attained 180 MPa with a curing temperature of 85 °C and duration of 30 h. The same strength level was achieved even in the cast-in-place components with a curing temperature of 40 or 60 °C maintained for 7 days. Honma *et al.* [17] compared the strength development of UHPC cured at 20 and 40 °C with that at 90 °C steam curing. The difference of strengths at each curing temperature was significant at 7 and 28 days, but became less significant at 91 days. The chemical mechanism and microstructure during curing and hydration of concrete are presented in some references [18,19].

## 3. Curing Tests of UHPC

### 3.1. Test Variables and Preparation of Specimens

As discussed previously, the quality of UHPC is largely affected by curing conditions, such as curing temperature and moisture condition, *etc.* However, preparing the steam curing system on a construction site (which is necessary for ensuring rapid strength development) would be uneconomical and involve some difficulties due to its temporary use during curing and the required movability along the casting place of concrete. Therefore, it would be very important to determine an efficient curing method for cast-in-place UHPC by taking into account the site condition, construction period, economy, and required strength of UHPC.

**Figure 3:**
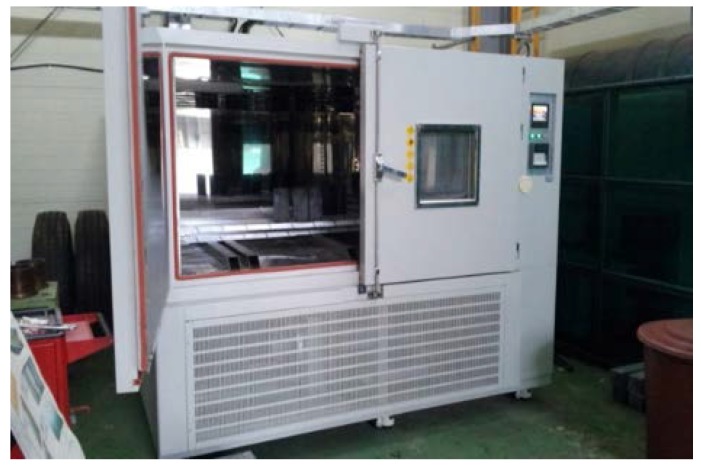
Constant temperature and humidity chamber.

In this study, the test variables are determined by relaxing the conditions of the prototype curing method of K-UHPC [4,13], which has been deonstrated to be sufficient for ensuring the specified compressive strength of 180 MPa immediately after curing. The prototype follows the order of initial moist curing for 24 h after casting, form removal, and steam moist curing with 90 °C for 48 h. As shown in Table 2, additional conditions considered in this study are: the lower curing temperatures of 20, 40 and 60 °C; the duration of the initial curing (also called delay time in this study) that is shortened to 12 h or extended to 48 h; the duration of the main curing (also called curing time or continuing time in this study) that is shortened to 12 or 24 h; and the type of moist curing conditions. The moisture conditions during curing are categorized into four types. The enclosed or sealed condition is realized by tightly wrapping the specimen with polyethylene sheet to ensure that the internal moisture does not evaporate. A dry condition is provided by a dry heating chamber, while a constant temperature and humidity chamber as shown in Figure 3 is used to apply the water or steam condition.

**Table 2:** Variables of curing test.

Curing Temperature (°C)	Delay Time (h)	Continuing Time (h)	Moisture Condition
20	24	12	enclosed, water
		24	
		48	
		72	
40	12	12	dry, enclosed, water, steam
		24	
		48	
	24	12	
		24	
		48	
	48	12	
		24	
		48	
60	12	12	
		24	
		48	
	24	12	
		24	
		48	
	48	12	
		24	
		48	
90	24	48	
		72	

The target curing temperature of the chamber is attained at a 15 °C increase per hour and the same rate of temperature variation is applied when descending. The curing time is evaluated based only on the period of constant temperature. Although moisture is continuously supplied during the initial curing period with the standard curing method of K-UHPC, considering any adverse site situation, it is assumed in this study that the specimen is subjected to a dry condition during the initial curing, regardless of the form removal conducted at 12 h after casting. Therefore, the initial curing period is also called the delay time in this study because the curing is not actually performed during this period. In the enclosed condition, however, the specimen is sealed immediately after the form is removed, as can be expected on site. Furthermore, while other moisture conditions last only as long as the curing time, the enclosed condition is maintained until the strength is measured at 7 days since this situation can be easily applied on site. Because the purpose of this study is to examine how closely the strength attains the specified compressive strength within an early age, 7-day compressive strengths are measured according to the standard test method [4,20]. The average compressive strength is calculated by averaging the strengths of the three specimens for each test variable. The shape of the specimen is a cylinder with 100 mm diameter and 200 mm height according to the relevant specifications [4,21]. As will be shown in the comparison provided in the later part, these are the most widely-used dimensions, as far as the cylindrical shape is concerned. Although a specimen with different size was used in some previous studies, the size did not exceed 150 mm in diameter and 300 mm in height at most. It can be sufficiently assumed that the internal temperature of these small-sized specimens used in practice, whether it is a cylinder or a cube, is uniformly distributed according to the ambient curing temperature; and, thus, the effect of the shape and size of a specimen on the temperature distribution and related strength development is negligible. The components of K-UHPC were mixed using a dedicated mixer that was developed for UHPC [8]. The test specimens were prepared by following the related specifications [4,21] in terms of placing, consolidation, finishing, and ensuring plane ends.

**Figure 4:**
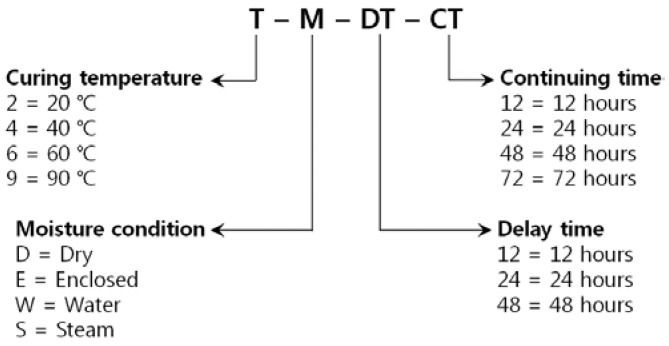
Abbreviations of variables of curing test.

The test variables of this curing test basically include four cases of curing temperature, three cases of delay time before the initiation of main curing, three cases of main curing time and four cases of moisture condition. These are summarized in Table 2 and Figure 4 with explanations for several abbreviations. In Figure 4, if one of the letters “T”, “M”, “DT” and “CT” is given as it is, then the entire test variables related to this letter are included in the corresponding case. For example, T-M-24-48 indicates the cases that are cured for 48 h starting from 24 h after casting, with all the curing times and moisture conditions included.

### 3.2. Effect of Curing Temperature

The average compressive strength of the specimens made with standard steam curing (24 h initial curing and subsequent 48 h main curing at 90 °C) was 201.0 MPa as shown in Table 3, which is 112% of the specified strength of 180 MPa. The test results, as affected by various curing temperatures, with other conditions remaining the same as those of the standard steam curing, are presented in Table 3. As the curing temperature decreased, the average compressive strengths also reduced, which results in 97%, 76% and 61% of the specified strength for the curing temperatures of 60, 40 and 20 °C, respectively.

**Table 3:** Compressive strengths according to curing temperatures (T-S-24-48).

Variable	Compressive Strength (MPa)			Average Compressive Strength (MPa)	Standard Deviation (MPa)
	#1	#2	#3		
2-W-24-48 *	116.7	110.2	101.0	109.3	6.44
4-S-24-48	142.3	133.6	133.0	136.3	4.25
6-S-24-48	179.9	170.9	171.4	174.1	4.13
9-S-24-48	200.6	201.5	200.8	201.0	0.39

* Water curing is provided instead of steam curing in this case.

**Figure 5:**
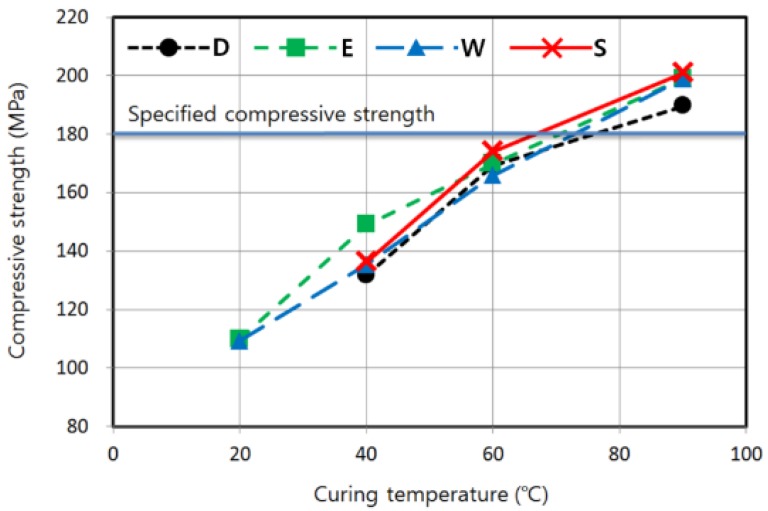
Compressive strengths according to curing temperatures (T-M-24-48).

Figure 5 shows the characteristics of strength development according to curing temperature and moisture condition with other conditions remaining the same. The compressive strength was proportional to the curing temperature, regardless of the moisture condition. Overall, the enclosed condition resulted in fairly good strength development when compared with other moisture conditions, especially at lower temperatures, although only passive measures were taken to prevent the evaporation of water in concrete. As mentioned previously, the enclosed condition was maintained until the strength was measured at 7 days, so that the remaining water in the concrete could be used for hydration and strength development. However, other moisture conditions were maintained only during the curing time, which means the specimens were exposed to a dry environment during the remaining time before strength measurement. As a result, for a curing temperature of 40 °C, the highest compressive strength was 149.0 MPa in the enclosed condition, while the lowest was 131.7 MPa in the dry condition. The strength obtained from the enclosed condition was approximately 10% higher than that of other moisture conditions for the curing temperature of 40 °C. Even for higher temperatures, the strength level in the enclosed condition was still high as it was only 2.4% and 1.0% lower than the highest strengths obtained in steam curing at 60 and 90 °C, respectively. Therefore, for strength development of K-UHPC, it is also effective to try to maintain the water contained in the concrete by wrapping the surface with a material such as polyethylene sheet instead of providing an active water supply to the concrete. In other words, when a continuous supply of water is not feasible, the next most effective strategy is to protect the surface from evaporation. A dry condition had an adverse effect on the strength in all ranges of temperature due to the evaporation of the water needed for hydration. 

Therefore, it is apparent that the strength development of K-UHPC is accelerated as the curing temperature increases. Although the 7-day strength of 60 °C presented in Figure 5 did not attain the specified compressive strength, the difference between these two strengths was not considerable. As will be shown later, in some other cases cured at 60 °C, the specified strength was exceeded by adjusting the delay time.

It is also important to estimate when the specified strength is attained for cases where the specified strength is not reached in 7 days. Several of the cases exceeded the specified strength at 28 days, as will be discussed later. Many of the other cases may eventually attain the specified strength, as can be seen in Figure 2 and as confirmed in previous studies. Whether or not the delayed strength development is acceptable depends on the progress schedule of the site and construction period.

Figure 5 shows an almost linear strength development for all moisture conditions. Therefore, the following predictive equations are proposed as a result of regression analysis that relate early-age compressive strength to curing temperature, representatively for enclosed and water conditions:

(1) $$f_{\text{c7}}/f_{\text{c7,}T\text{=90}}=0.0062T+0.4627\text{ (20 °C ≤ }T\text{ ≤ 90 °C, for enclosed condition)}$$

(2) $$f_{\text{c7}}/f_{\text{c7,}T\text{=90}}=0.0065T+0.4246\text{ (20 °C ≤ }T\text{ ≤ 90 °C, for water condition)}$$

where *f*_c7_ is the 7-day compressive strength (MPa), *f*_c7, *T* = 90_ is the 7-day compressive strength for a 90 °C curing temperature (MPa), and *T* is curing temperature (°C). These equations are normalized with respect to *f*_c7, *T* = 90_. The coefficients of determination (*R*^2^) of Equations (1) and (2) are as high as 0.9675 and 0.9955, respectively, which indicates that linear regression can provide a sufficiently reliable estimation of the strength. Similarly, such a predictive equation can be proposed for other cases of delay time, curing time, and moisture conditions.

### 3.3. Effect of Curing Time

**Figure 6:**
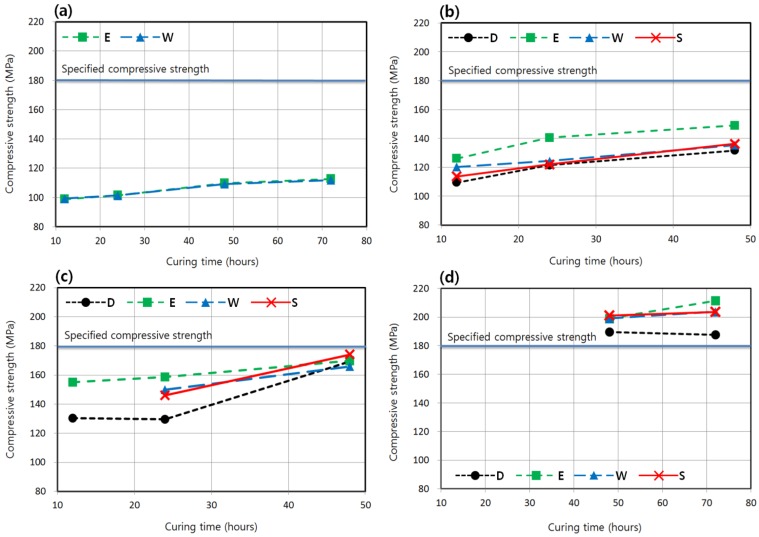
Compressive strengths according to curing times (T-M-24-CT). (**a**) Curing temperature = 20 °C (2-M-24-CT). (**b**) Curing temperature = 40 °C (4-M-24-CT). (**c**) Curing temperature = 60 °C (6-M-24-CT). (**d**) Curing temperature = 90 °C (9-M-24-CT).

The effect of increasing the length of time of the main curing on strength development is investigated in this section. Figure 6 shows the average strengths of specimens as affected by curing time that were cured at each temperature with the delay time fixed as 24 h. For this delay time, regardless of moisture condition, while the specimens of 7-day strength cured at 90 °C exceeded the specified strength, the specimens cured at lower temperatures did not reach the specified strength of 180 MPa, even with a curing time of 48 h. However, the specimens that were cured at 60 °C almost attained the specified strength, as shown in Figure 6c. Therefore, it would be possible for these specimens to reach the specified strength with some measures, such as a slightly increased curing time and an adjustment of the delay time, as will be presented later.

In the case of specimens cured at 20 °C, the strength increase according to curing time was so marginal that the specified strength could not be attained within 7 days. Referring to the previous study on K-UHPC shown in Figure 2, when applying a temperature of 20 °C, the specified strength can only be ensured in the long term with a continuous supply of moisture. The strengths obtained from enclosed and water conditions at each curing time do not significantly differ in the case of 20 °C. As mentioned previously, the enclosed condition that can be relatively easily realized on site provides a better treatment of moisture and strength development in this study, regardless of curing temperature, as shown in Figure 6. Although the specified strength was not reached at 40 and 60 °C with the curing times considered, a regression equation can be used to estimate the appropriate curing time if the specified strength is to be ensured in 7 days. For the enclosed condition that shows a better strength development, it appears that the linear regression can provide the best fit, resulting in the following equations:

(3) $$f_{\text{c7}}=0.5972t+121.82\text{ (for curing temperature of 40 °C)}$$

(4) $$f_{\text{c7}}=0.4158t+149.57\text{ (for curing temperature of 60 °C)}$$

where *f*_c7_ is the 7-day compressive strength (MPa) and *t* is the curing time (h). *R*^2^ of Equations (3) and (4) are 0.8885 and 0.9907, respectively, with sufficient accuracy. According to Equations (3) and (4), the specified strength can be ensured in 7 days with the curing time of 97 and 73 h, *i.e.*, approximately 4 and 3 days, for curing temperatures of 40 and 60 °C, respectively. Matsubara *et al.* [6] stated that 180 MPa was attained after 7 days curing at 40 or 60 °C. Consequently, the K-UHPC of this study shows a better strength development performance even with a shorter curing time when compared with the UHPC developed by Matsubara *et al.* [6]. At 90 °C, increasing the curing time from the standard 48 to 72 h was also effective, except for the dry condition. In contrast, for the dry condition, the strength slightly decreased due to the excessive evaporation of internal water which induced drying and micro-cracks under a very high temperature [13].

**Figure 7:**
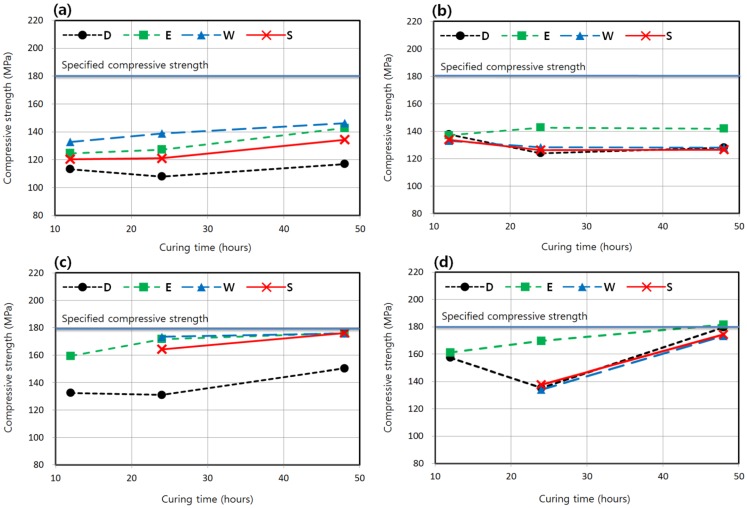
Compressive strengths according to curing times (T-M-DT-CT). (**a**) Curing temperature = 40 °C and delay time = 12 h (4-M-12-CT). (**b**) Curing temperature = 40 °C and delay time = 48 h (4-M-48-CT). (**c**) Curing temperature = 60 °C and delay time = 12 h (6-M-12-CT). (**d**) Curing temperature = 60 °C and delay time = 48 h (6-M-48-CT).

Figure 7 shows the relationship between strength and curing time for delay times other than 24 h. At a shorter delay time of 12 h, which corresponds to the immediate initiation of curing after form removal, the strength was higher in the water condition than that in the enclosed condition, especially for that shown in Figure 7a at 40 °C. It is because the advantage of the enclosed condition is less distinct when the delay time is short. However, the enclosed condition resulted in better strengths in the delay time of 48 h (Figure 7b,d) than other moisture conditions, as did in the delay time of 24 h (Figure 6b,c). As shown in Figure 7b, at a curing temperature of 40 °C, the longer delay time of 48 h meant that the effect of increasing the curing time on strength development was less clear. Figure 6c and Figure 7c,d show that the strength development at 60 °C curing in the enclosed condition is quite satisfactory; the specified compressive strength can almost be reached or can even be exceeded in 7 days with the curing time of 48 h adopted in the standard steam curing of K-UHPC. On the other hand, it is estimated that at a curing temperature of 40 °C, a few days curing time may be needed to reach the specified strength in 7 days, as shown in Figure 6b and Figure 7a.

### 3.4. Effect of Delay Time

According to a previous study on the standard steam curing of K-UHPC [13], if the delay time before high-temperature curing of 90 °C is too short, strength degradation may possibly occur because cement hydrate is exposed to a high temperature before it hardens to form a tight structure and is prone to internal micro-cracks. The reason why the delay time or the initial curing period before the main steam curing is set at 24 h is based on this previous study [13]. Honma *et al.* [17] demonstrated that if the high-temperature curing initiates after the initial setting that corresponds to the penetration resistance of 5 MPa, similar strengths are obtained regardless of the delay time in the UHPC with the specified compressive strength of 150–200 MPa. In comparison, the initial setting of concrete mixture is defined by the time when the penetration resistance equals 3.5 MPa according to ASTM C403 [22]. Ahlborn *et al.* [14] reported that a delay time as long as 10 or 24 days before high-temperature curing at 90 °C induced a slight decrease of strength. However, the delay times of this study are the shortest at 12 h when the initial setting time has already passed, and are the longest at 48 h, which are not as long as the delay time of the study by Ahlborn *et al.* [14]. Furthermore, the curing temperatures that are the main focus in this study are 40 and 60 °C, which are lower than the curing temperature of 90 °C adopted in the above previous studies carried out on the effect of the delay time. As a result, the effect of the delay time on strength development is not distinct in this study.

**Figure 8:**
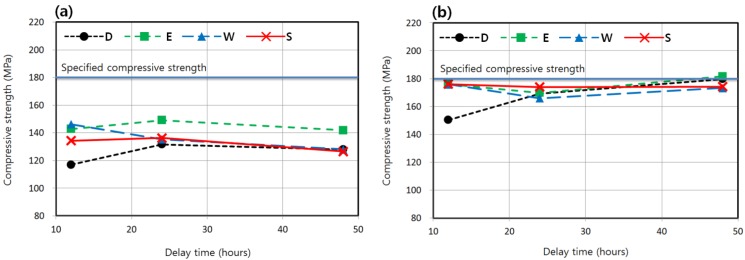
Compressive strengths according to delay times before curing (T-M-DT-48). (**a**) Curing temperature = 40 °C (4-M-DT-48). (**b**) Curing temperature = 60 °C (6-M-DT-48).

As seen in Figure 8, with the curing time of 48 h adopted for standard steam curing, and with the curing temperature of 40 or 60 °C, the effect of the delay time ranging from 12 to 48 h on the strength was not clear. That is, increasing the delay time did not result in a consistent increase or decrease of the strength. 

### 3.5. Discussion

The results of this study and the relevant previous studies are compared in Table 4. The specified compressive strengths of the UHPC are considered to be in the range from 150 to 200 MPa. The comparison is limited to the lower curing temperatures, ranging from 20 to 70 °C, than usual steam curing temperature of 90 °C. Also, the development of early-age strength of less than or equal to 7 days is compared, according to the focus of this study. It can be identified that this study took more variables or parameters than the previous studies to investigate various aspects regarding the curing of UHPC. It is notable that in a few studies including this study, the specified compressive strength could be attained in as early as 7 days, with careful consideration of the temperature and duration of the curing. In order to achieve this purpose, at least 40–60 °C was required as curing temperature, whereas room temperature at around 20 °C was insufficient. The shape and size of the specimens of various studies are also compared in Table 4.

In addition to the early-age strengths measured at 7 days, 28-day strengths were also measured, although they are not the main focus of this study. Some of the specimens that were cured at 60 °C and that closely approached the specified compressive strength at 7 days exceeded the specified strength at 28 days with the strength increase of 4.3% on average, which included 6-E-12-48, 6-W-12-48 and 6-S-12-48. Other specimens required a longer term to attain the specified strength. When comparing the results of strength measurement among the different studies, the length of time the curing was maintained should be noted to ensure a reasonable comparison. For a long-term strength measurement at 28 days or later, some studies applied moist curing continuously until the strength was measured [3,13], while other studies, including this study, maintained moist curing only for a short period [1]. The UHPC developed by Wille *et al.* [3] attained 190–200 MPa at 28 days when continuously stored in water at 20 °C and the K-UHPC of this study reached 190 MPa at 91 days in a similar condition [13]. The UHPC developed in the Sustainable and Advanced Materials for Road InfraStructure (SAMARIS) project [1] achieved 182 MPa at 28 days in ambient temperature with 8 days of moist curing, although around 150 MPa was attained at 7 or 14 days. However, the previous studies mentioned above focused on a long-term strength of at least 28 days; therefore, because this study deals with the early-age strength of UHPC at 7 days, it can provide distinct and useful data for UHPC cast in-place for field application.

**Table 4:** Comparison of the test results with previous studies on UHPC.

Study	Variables	Shape and Size of Specimen (mm)	Specified Compressive Strength (MPa)	Water-to-Binder Ratio/Mineral Admixture (% of Binder)	Early-Age Strength Development (Curing Condition)
This study	Curing temperature, delay time, continuing time, moisture condition	Cylinder (Φ 100 × 200)	180	0.2/Silica fume (20%)	180 MPa in 7 days (40 °C for 4 days or 60 °C for 2 days in moist condition)
SAMARIS [1]	-	Cylinder (Φ 110 × 220)	180	0.123/Silica fume (21%)	150 MPa in 7 days (20 °C for 7 days in moist condition)
Ishii *et al.* [5]	Curing temperature	Not specified	180	Not specified	147 MPa in 4 days (70 °C for 2 days)
Matsubara *et al.* [6]	Curing temperature	Cylinder (Φ 100 × 200)	180	0.152/Silica fume (not specified)	180 MPa in 7 days (40 °C or 60 °C for 7 days)
Ahlborn *et al.* [14]	Curing temperature, delay time	Cylinder (Φ 76 × 152)	200	Not specified	137 MPa in 7 days (20 °C for 7 days)
Nakayama *et al.* [16]	Curing temperature, delay time, continuing time	Cylinder (Φ 100 × 200)	150	0.15/Silica fume (15%)	135 MPa in 7 days (60 °C for 3 h in moist condition)
Honma *et al.* [17]	Curing temperature	Cylinder (Φ 100 × 200)	150–200	0.12–0.2/Silica fume (10%–20%)	90–100 MPa in 7 days (20 °C for 7 days in moist condition) and
					130–170 MPa in 7 days (40 °C for 7 days in moist condition)

According to the experimental results of this study, it would not be possible to ensure the specified compressive strength of K-UHPC within 7 days with a curing temperature of 20 °C, regardless of the moist curing method employed. The minimum condition to ensure the specified strength at 7 days derived in this study is a curing period of 48 h (2 days) with a temperature of 60 °C under a moist condition, and with the delay time of 12 to 48 h before the curing begins. A maximum of 3 days curing period would be sufficient to ensure the specified strength in 7 days. A certain type of heat treatment would be required to ensure a curing temperature of 60 °C, such as a heating system that is easily available on site as adopted by Matsubara *et al.* [6] in an actual pedestrian bridge. On the other hand, at a curing temperature of 40 °C, at least a 4 days curing period is required to attain the same strength level. In the hot weather conditions in some countries, an environmental temperature of 40 °C may be obtained simply by covering the structure with a thick and tight plastic sheet after casting, even though a heating facility is not available on site.

## 4. Conclusions 

This study presented experimental results on the effect of various curing conditions of K-UHPC on early-age strength development in order to increase the field applicability of K-UHPC. Based on the results of the foregoing investigation, the following conclusions can be drawn:

- As the curing temperature increased, the development of the compressive strength of K-UHPC was accelerated. The 7-day strength was almost linearly proportional to the curing temperature, which ranged from 20 to 90 °C. Some specimens cured at 60 °C for 48 h attained 180 MPa in 7 days, which is the specified compressive strength of K-UHPC, under the condition that proper delay time along with careful moisture treatment are provided. A dry condition should be avoided, especially in UHPC based on a low water-to-binder ratio. The enclosed condition where the concrete surface is covered with a thin plastic sheet was also effective when compared with moist curing.
- The strength development of K-UHPC was also proportional to the curing period, regardless of the curing temperature. In order to ensure the specified strength in 7 days, the curing time of 48–72 h was appropriate for a curing temperature of 60 °C, while a longer period of at least 96 h may be necessary for 40 °C based on the regression analysis. However, the specified strength is not attained in 7 days if a curing temperature of 20 °C is maintained even until strength measurement.
- The effect of delay time ranging from 12 to 48 h prior to initiation of the main curing on the strength development of K-UHPC was not clear, regardless of the curing temperature. In field application, therefore, the delay time would not have an adverse effect on the strength unless it is either too long or too short.
